# Variations in Ionospheric Parameters Associated With the 20–21 May 2012 Annular and 21 August 2017 Total Solar Eclipses

**DOI:** 10.1155/tswj/5547996

**Published:** 2026-03-11

**Authors:** Manghang Limbu, Samyam Pudasaini, Sujan Prasad Gautam, Binod Adhikari, Ashok Silwal, Iva Kumari Lamichhane, Rohit Bhattarai, Pitri Bhakta Adhikari

**Affiliations:** ^1^ Department of Physics, Tri-Chandra Multiple Campus, Kathmandu, Nepal; ^2^ Department of Space Science, University of Alabama in Huntsville, Huntsville, Alabama, USA, uah.edu; ^3^ Depeartment of Physics, Patan Multiple College, Tribhuvan University, Lalitpur, Nepal, tribhuvan-university.edu.np; ^4^ Space Research Centre, Nepal Academy of Science and Technology, Lalitpur, Nepal, nast.gov.np

**Keywords:** annular eclipse, ionosphere, solar storm indices, total eclipse, total electron content

## Abstract

This study investigates the ionospheric effects of the annular solar eclipse (20–21 May 2012) and total solar eclipse (21 August 2017) across midlatitude stations, analyzing critical parameters: F2‐layer critical frequency (foF2), peak height (hmF2), and total electron content (TEC). Using ionosonde data and geomagnetic indices, we isolated eclipse‐driven perturbations by comparing observations with quiet‐day baselines. Both eclipses induced significant reductions in foF2 and TEC (~30%–38% during totality and 15%–25% during annularity), with pre‐eclipse transient enhancements, which might be attributed to traveling ionospheric disturbances (TIDs). The total eclipse caused a notable hmF2 uplift (~20 km), whereas the annular eclipse showed negligible changes, reflecting different thermospheric cooling magnitudes for eclipses with different magnitude and obscuration rates. Stations under higher obscuration (e.g., Idaho, 100%) experienced stronger depletions, supporting the role of eclipse magnitude. Recovery times for foF2 (1–3 h) exceeded TEC restoration, which might be due to transport‐driven F2‐layer dynamics. Although noticeable variation is observed on foF2 and TEC parameters, their magnitudes are found to vary with local time, latitude, and obscuration rate. These findings emphasize the ionosphere′s layered sensitivity to solar forcing and have implications for GNSS and communication systems vulnerable to TEC fluctuations.

## 1. Introduction

Solar eclipses are natural phenomena that occur when the Moon, Sun, and Earth align in a straight line [1]. These events are categorized into different types, for example, annular solar eclipses, where the Moon′s apparent diameter is smaller than the Sun′s diameter, leaving a visible “ring of fire” (called annulus) around its shadow [2], and total solar eclipses, during which the Sun is entirely obscured by the Moon. The abrupt reduction in solar irradiance during these events disrupts Earth′s ionosphere, a layer of the upper atmosphere ionized by solar radiation, by reducing photoionization processes, leading to measurable declines in electron density [1, 3]. This ionospheric perturbation serves as a unique natural experiment to study atmospheric responses to sudden changes in solar forcing [4].

Ionospheric responses to eclipses exhibit significant spatiotemporal variability. The Moon′s umbral shadow traverses the ionosphere at supersonic speeds (> 3000 km/h) [5], creating rapid fluctuations in solar ionizing flux. Lower ionospheric layers (E and F1), governed by photochemical equilibrium, show immediate electron density drops due to suppressed extreme ultraviolet (EUV) radiation [6, 7]. In contrast, the F2 layer′s response is modulated by slower transport processes like ambipolar diffusion and thermospheric winds, leading to delayed and location‐dependent effects [8, 9]. Midlatitude regions often exhibit posteclipse plasma uplift due to wind‐driven redistribution [10], whereas equatorial zones may experience enhanced plasma instability [11].

The ionosphere′s sensitivity to eclipses is reflected in various parameters such as the F2‐layer critical frequency (foF2), the height of peak electron density (hmF2), and total electron content (TEC). These parameters vary depending on the eclipse′s duration, magnitude, geographic coverage, and the Sun–Earth–Moon geometry [12–14]. For instance, annular eclipses reduce solar EUV flux, cooling the thermosphere‐ionosphere system and lowering electron density in the E, F1, and F2 layers to moderate levels compared with total solar eclipse [4, 15]. It is also important to note that separating eclipse‐induced effects from geomagnetic activity is crucial, and it requires careful analysis of indices like Disturbance Storm Time (Dst), Auroral Electrojet (AE), SYM/H, and Kp to rule out storm‐driven disturbances [16]. This is because the geomagnetic storms, triggered by solar wind interactions, can influence eclipse effects by altering ionospheric dynamics through particle precipitation and Joule heating [17, 18].

The thermal and dynamical consequences of eclipses further complicate ionospheric behavior. The sudden loss of solar heating creates a “cold spot” in the atmosphere, driving acoustic‐gravity waves (AGWs) that propagate vertically and horizontally [19, 20]. Posteclipse, the gradual restoration of solar flux reheats the thermosphere, restoring electron density through recombination and photoionization. These dynamics are observable as transient TEC depletions [21], geomagnetic pulsations [22], and traveling ionospheric disturbances (TIDs) [23].

To quantify these disturbances, researchers employ various methodologies. These include Faraday rotation to assess electron content variations, ionosonde networks to monitor layer‐specific plasma dynamics [12, 13], incoherent scatter radars to resolve altitude‐dependent ionization profiles [24], and GNSS‐derived TEC measurements to capture integrated electron density changes [4, 25, 26]. Prior studies have also leveraged multi‐instrument approaches. For example, Walker et al. [27] combined ionosonde, magnetometer, and infrasound data to analyze the 1988 East Asian eclipse, whereas Monani et al. [28] integrated GPS, incoherent scatter radar, and geomagnetic field observations during the 2008 Northern Hemisphere event. Till present, numerous advancements in the understanding of ionospheric response to the eclipses have been made. For instance, recent studies have revealed that lunar shadow dynamics alter thermospheric wind patterns, inducing vertical plasma redistribution: downward transport during obscuration and upward motion posteclipse [8, 10]. These asymmetric responses show the competing roles of photochemical loss, ambipolar diffusion, and wind‐driven transport, modulated by geographic and geomagnetic factors [29, 30].

In this study, we focused on midlatitude ionospheric responses. We used observations from ionosonde data at midlatitude stations to examine the impact of total and annular solar eclipses on the ionosphere directly above the radar location. The ionospheric parameters, foF2, hmF2, and TEC at the five American ionosonde stations for the total solar eclipse of 21 August 2017 and four American and Asian ionosonde stations for the annular eclipse of 20–21 May 2012 are analyzed. The ionospheric parameters are compared between the days of the eclipses and the geomagnetically quietest day of the same month. Geomagnetic storm indices were also analyzed to confirm that geomagnetic activity did not influence the ionosphere during the eclipse.

## 2. Data Sources

**Figure 1:**
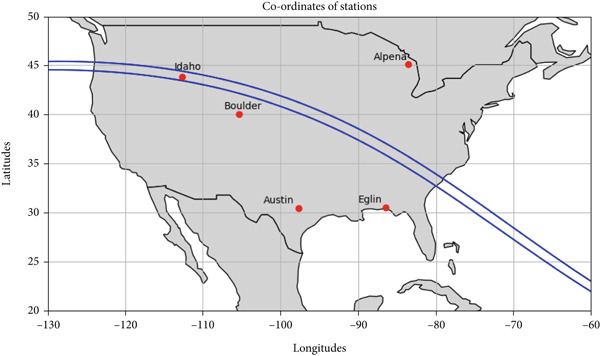
Map of Total Solar eclipse shadow path (area between two blue curves indicates region where total coverage of the sun occurred) and stations in North America on 21 August 2017. X and Y axes show geographical longitude and latitude, respectively. Red circles are the location of the stations.

**Figure 2:**
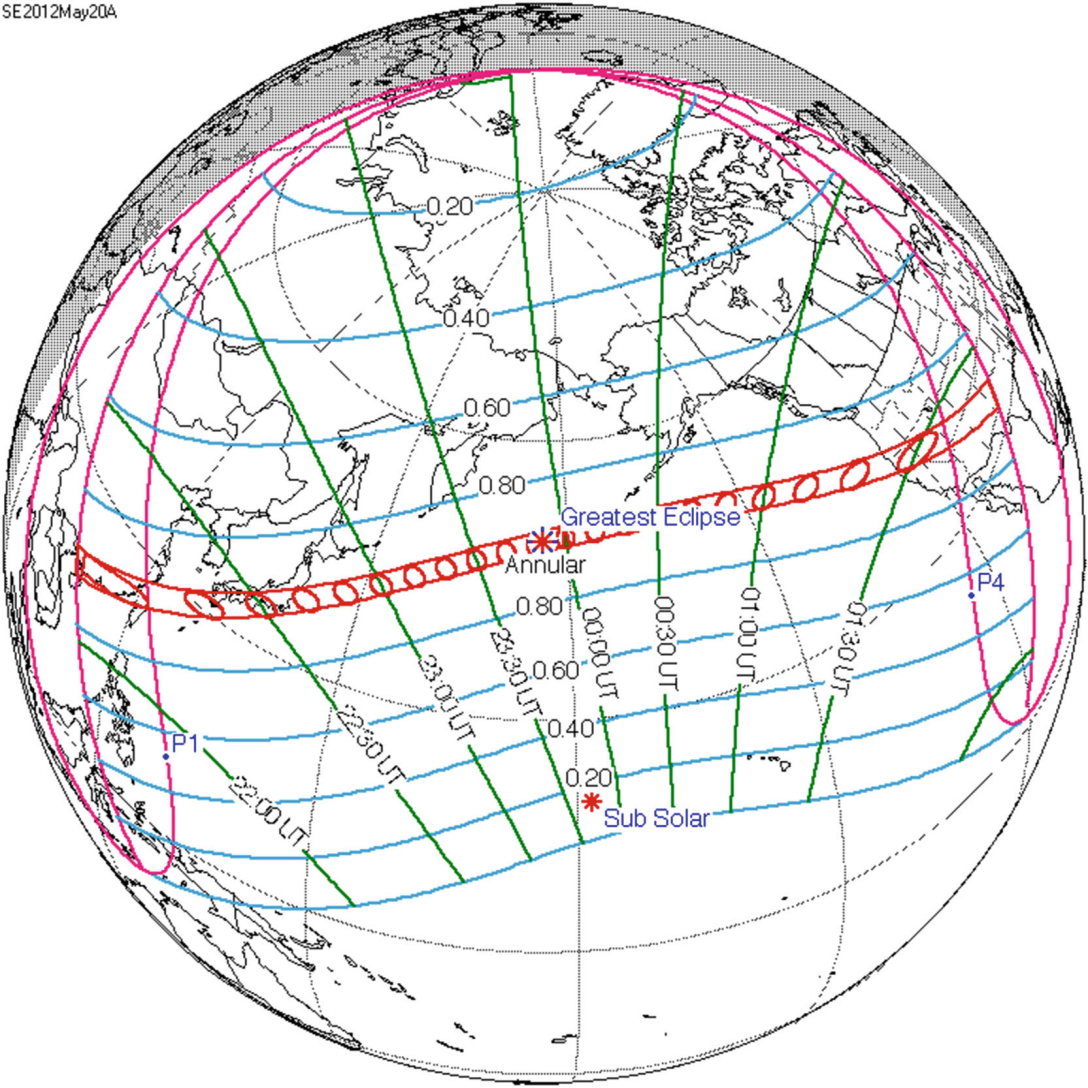
Map of annular solar eclipse shadow path and stations in Eastern Asia on 21 May and in North America on 20 May 2012 (courtesy of NASA). The red lines cover the path of the total solar eclipse and green lines separate the time difference (Universal Time) at different geographical locations.

We obtained F2‐layer parameters, critical frequency (foF2), peak height (hmF2), and ionogram‐derived TEC, from a chain of ionosondes located at Idaho (43.81° N, 112.68° W), Boulder (40.00° N, 105° W), Eglin (30.50° N, 86.5° W), Alpena (45.07° N, 83.56° W), Jeju (33.43° N, 126.30° E), and I‐Cheon (37.14° N, 127.54° E). Figure 1 displays the map of ionospheric stations during the total solar eclipse on 21 August 2017, with blue curves marking the umbra region where the sun was fully obscured. The eclipse path crosses the northwest region to southeast region of the United States. We considered five different stations in the United States with different obscuration rates. Idaho fell within the total eclipse zone (100% coverage), whereas Boulder, Eglin, Alpena, and Austin experienced partial eclipses with solar coverage of 93.1%, 83.04%, 71.76%, and 65.76%, respectively. Figure 2 shows a similar map for the annular solar eclipse on 20–21 May 2012, with red curves representing the shadow path and red circles illustrating the shape of the Moon′s shadow. As shown in the figure, the eclipse traced a path across parts of eastern Asia and continued across the Pacific Ocean before reaching the western United States. Regions within the red central band experienced a full annular eclipse, whereas a broader area, including much of North America, Southeast Asia, northern Australia, and parts of the Arctic, witnessed a partial eclipse. We considered four stations: Jeju (33.43° N, 126.30° E), I‐Cheon (37.14° N, 127.54° E), Idaho, and Boulder, recording coverage of 81.8%, 75.01%, 76.85%, and 78.99%, respectively.

The foF2, hmF2, and TEC data were sourced from the National Oceanic and Atmospheric Administration (NOAA) (https://www.ngdc.noaa.gov/stp/iono/). Geomagnetic storm indices at 1‐h resolution were retrieved from the OMNI Database (https://omniweb.gsfc.nasa.gov/form/dx1.html), whereas 5‐min SYM/H data were obtained from the High‐Resolution OMNI site (https://omniweb.gsfc.nasa.gov/form/omni_min.html) and averaged to 1‐h resolution. Eclipse‐related information, including event timing and path geometry, was gathered from Time and Date (https://www.timeanddate.com/eclipse/) and NASA′s eclipse archive (https://eclipse.gsfc.nasa.gov/). For comparative purposes, the quietest geomagnetic days of the same months in which the eclipses occurred were selected based on the criteria from the Kyoto World Data Center (https://wdc.kugi.kyoto-u.ac.jp/qddays/). We follow the method of Gautam et al. [26] to derive the quiet‐day value. For that, we calculated the median values of foF2, hmF2, and TEC during the five quietest days of the eclipse month. Finally, we subtract the quiet‐day values from the eclipse‐day values at each time interval to derive the ionospheric changes caused by the eclipse.

## 3. Results and Discussion

### 3.1. Event 1: 21 August 2017

**Figure 3:**
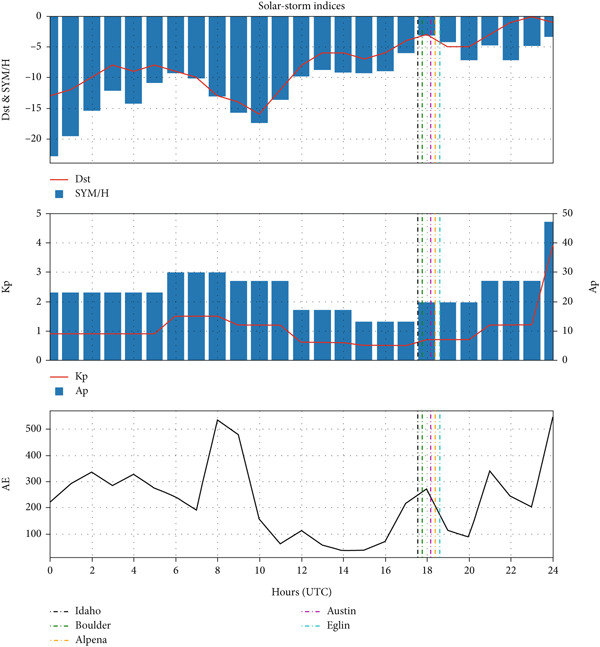
Variation in Dst (nT) and SYM/H (nT) indices in the top panel, Kp and Ap (nT) indices in the middle panel, and AE index (nT) in the bottom panel during total solar eclipse of 21 August 2017. The vertical lines denote the time of eclipse maxima at each station.

Figure 3 shows the geomagnetic indices during the total solar eclipse of 21 August 2017. The maximum of the solar eclipse occurred at 5:33 PM in Idaho, 5:46 PM in Boulder, 6:37 PM in Eglin, 6:23 PM in Alpena, and 6:10 PM in Austin, which is also indicated by the vertical dotted lines in the figure. The plots have clearly shown that Dst and SYM/H indices were at their minimum during the main phase of the eclipse rather than at the fore or later hours of the eclipse. All the stations, when shadowed through the eclipse event, noted a Dst index of nearly −3nT, which is very much less than the threshold Dst required for a storm to take place. Since the AE index and Kp index also measure very low magnitudes (∼250nT and 2 [Kp], respectively) during the eclipse, it is obvious that there was no storm event on the day. Therefore, the eclipse day was a quiet day and that is why all the changes brought in the ionospheric parameters that day are mostly caused by the eclipse.

Figure 4 shows a comparison of the changes in foF2 during the quiet day as a green curve and the eclipse day as a red curve. We can observe a clear diurnal pattern on the foF2 parameter, that is, smaller during the morning/nighttime and larger during the daytime. The foF2 parameter also has a similar trend as TEC. We expect that the value of TEC at the time of eclipse, thus, like TEC, foF2 also decreases during the eclipse period, as it is observed in the figure. We also see that the value of foF2 and TEC is higher at Austin and Eglin, which are at the lowest latitudes. This effect can correspond to the increase in the n(O)/n(N2) ratio in lower latitudes in the midlatitude region [31–33]. Before the onset of the eclipse there is a slight increase followed by a sharp decrease in foF2 in every station. The value of foF2 during an eclipse is lower than the quiet day as expected. There is a decrease in foF2 from ~5 MHz to ~4 MHz in Alpena, ~6.1 MHz to ~4.4 MHz in Austin, ~5.2 MHz to ~3.6 MHz in Boulder, ~5.5 MHz to ~4.5 MHz in Eglin and ~4.4 MHz to 3.8 MHz in Idaho during the eclipse period. The Idaho and Boulder stations are located near the path of totality, whereas Austin, Eglin, and Alpena are situated farther from it. Interestingly, the larger depletion rate was observed at Austin, which is away from the path of totality. This distribution may be influenced by the local time of eclipse occurrence. For instance, the eclipse occurred around 11:30 LT in Idaho and Boulder (see Table 1), whereas in Austin, it occurred after approximately 13:00 LT, a time when diurnal TEC tends to increase due to enhanced photoionization (see Figure 4, green curve). The pattern of decreasing foF2 after the onset of the eclipse is seen in every station up to the point of total coverage and an increase of foF2 is seen after total coverage. The rise of foF2 is seen after the eclipse in all five stations. We observed a posteclipse effect on the foF2 parameter in all stations, which is due to the time required by the ionosphere to settle down into the normal ionospheric condition.

Figure 4Changes in F2‐layer critical frequency (foF2) in MHz recorded at different stations, (a) Alpena, (b) Austin, (c) Boulder, (d) Eglin, and (e) Idaho, during total solar eclipse of 21 August 2017. The three vertical black lines indicate the beginning (left line) and the end (right line) of partial eclipse and maximum (middle line) of the solar eclipse.

**Figure:**
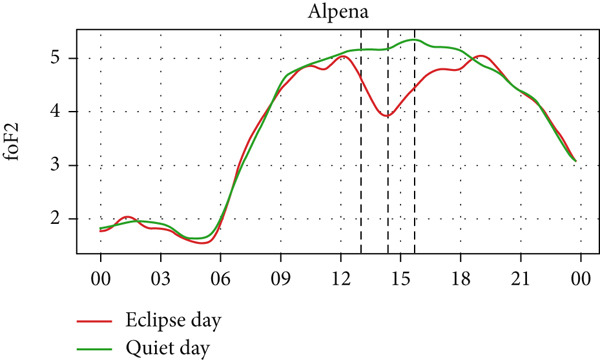
(a)

**Figure:**
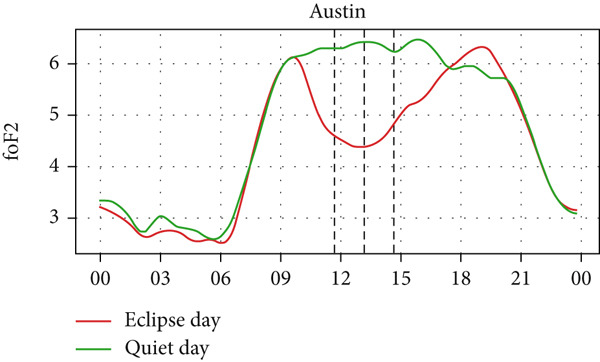
(b)

**Figure:**
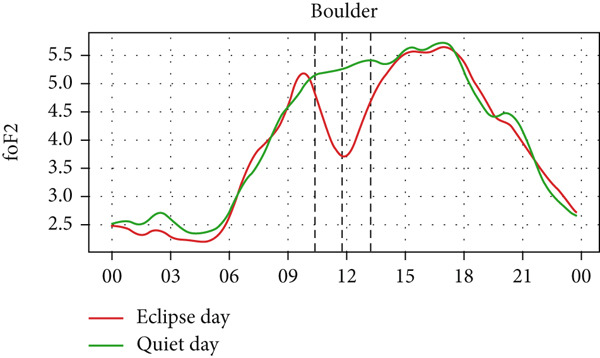
(c)

**Figure:**
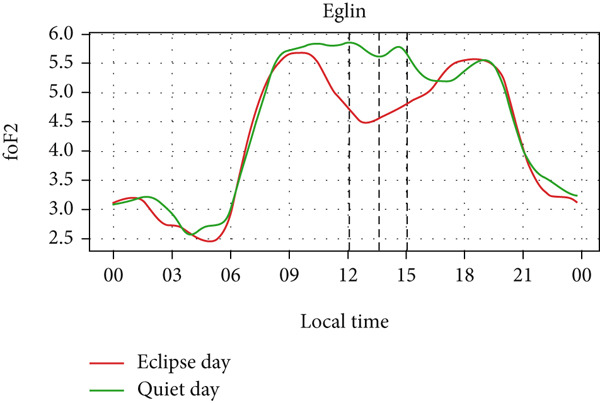
(d)

**Figure:**
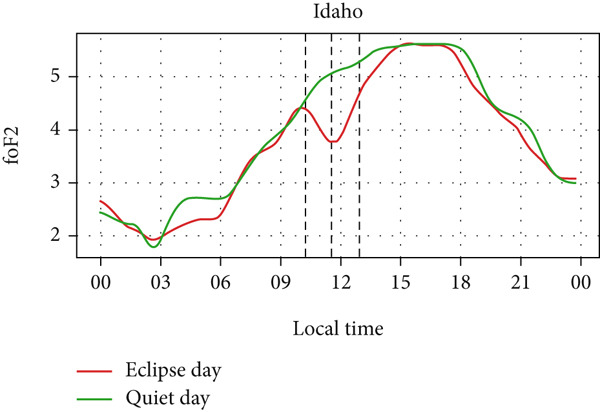
(e)

**Table 1:** From left to right, showing the number of stations, ionosonde station names, abbreviations, their geographical locations, and eclipse percentages.

**S.N.**	**Name of station**	**Station code**	**Latitude (degree)**	**Longitude (degree)**	**Eclipse percentage**
1.	Alpena	AL945	45.07	276.44	71.76%
2.	Austin	AU930	30.40	262.30	65.75%
3.	Boulder	BC840	40.00	254.70	93.1% (T), 78.99% (A)
4.	Eglin	EG931	30.50	273.50	83.04%
5.	I‐Cheon	IC437	37.14	127.54	75.01%
6.	Idaho	AC843	43.81	247.32	76.85% (A)
7.	Idaho	IF843	43.81	247.32	100% (T)
8.	Jeju	JJ433	33.43	126.30	81.08%

In Figure 5, we compare the TEC variation between the eclipse‐day and quiet‐day conditions. As anticipated, TEC values during the eclipse fell below quiet‐day baselines at all stations, reflecting diminished solar irradiance during an eclipse. Significant TEC declines coincided with the eclipse onset in Alpena, Boulder, and Idaho, whereas Austin and Eglin exhibited pre‐eclipse reductions. Quantitatively, TEC decreased by ~29% in Alpena (7.0⟶5.0 × 10^16^ m^−2^), ~30% in Austin (10.0⟶7.0 × 10^16^ m^−2^), ~34% in Boulder (6.5⟶4.3 × 10^16^ m^−2^), ~18% in Eglin (8.5⟶7.0 × 10^16^ m^−2^), and ~38% in Idaho (6.8⟶4.2 × 10^16^ m^−2^). Posteclipse recovery phases restored TEC to pre‐event levels across all sites. Stations with higher obscuration (Boulder: 93% and Idaho: 100%) experienced the most severe depletions (4.3 × 10^16^ m^−2^ and 4.2 × 10^16^ m^−2^, respectively), aligning with the eclipse‐driven suppression of solar EUV flux, which is a key driver of thermospheric cooling and ionospheric plasma loss [4]. In the past, latitudinal trends in TEC response were frequently reported, with high‐latitude reductions (10%–20%) [28, 34, 35], midlatitude depletions (30%–40%) [20, 36], whereas equatorial regions exhibited amplified reductions (> 40%), which is possibly due to equatorial‐anomaly dynamics [37], with a time lag of about 5–60 min between maximum TEC depletion and maximum obscuration time [26]. These observations show the altitude‐dependent interplay between photochemical processes (dominant in E/F1 layers) and transport mechanisms (governing F2‐layer variability) during eclipses [38, 39]. However, in our case, the observed TEC depletion varied with the obscuration rate rather than latitude. For example, the Boulder and Idaho stations, which are located near the path of totality, exhibited larger TEC depletion rates. In contrast, Austin and Alpena are situated farther from the path of totality, one at a higher latitude and the other at a lower latitude, but both experienced nearly equal distances from the totality path and similar depletion rates. That said, it should also be noted that the number of stations used in this study is small. For a statistically valid conclusion regarding the latitudinal effect, a larger number of stations should be considered. Pre‐eclipse TEC enhancements are also seen at stations with lower obscuration rates, potentially linked to TIDs [40], further indicating the complex wave‐driven interactions preceding obscuration, which is also frequently observed by some previous studies (e.g., [15, 26, 40]).

Figure 5Changes in TEC in (×10^16^ m^−2^) recorded at different stations, (a) Alpena, (b) Austin, (c) Boulder, (d) Eglin, and (e) Idaho, during total solar eclipse of 21 August 2017. The three vertical black lines indicate the beginning (left line) and the end (right line) of the partial eclipse and maximum (middle line) of the solar eclipse.

**Figure:**
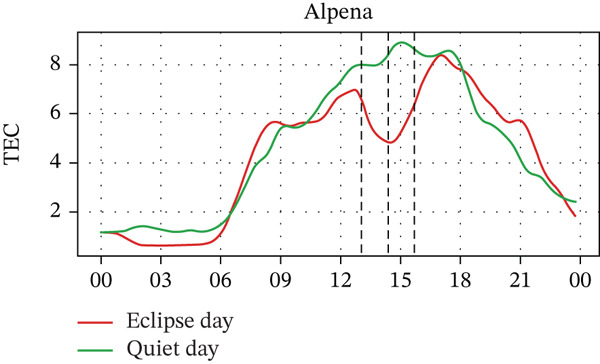
(a)

**Figure:**
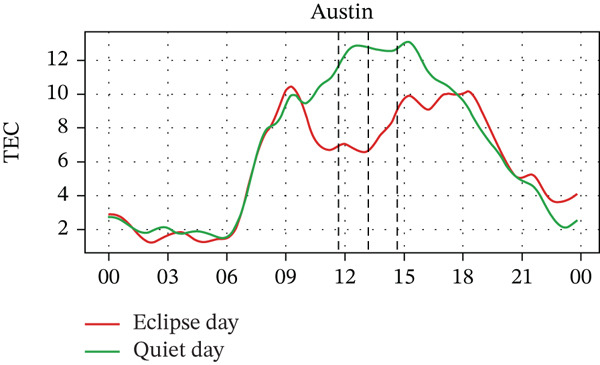
(b)

**Figure:**
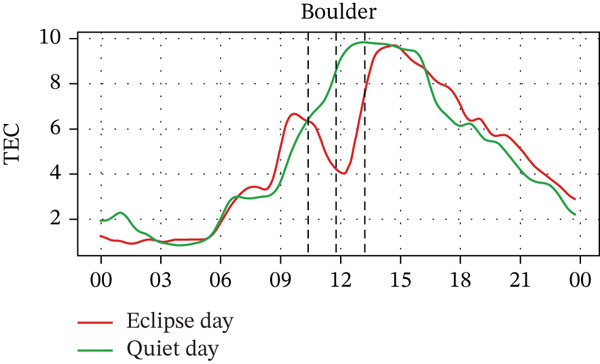
(c)

**Figure:**
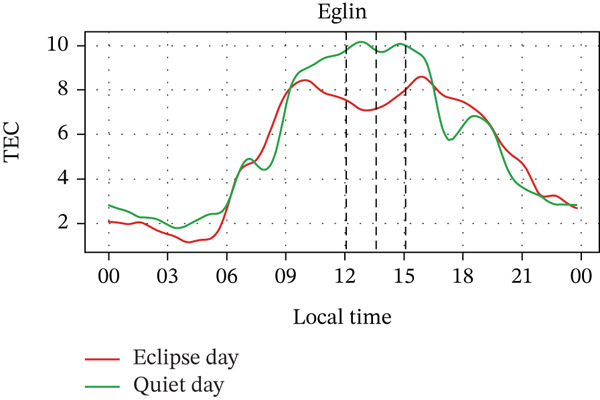
(d)

**Figure:**
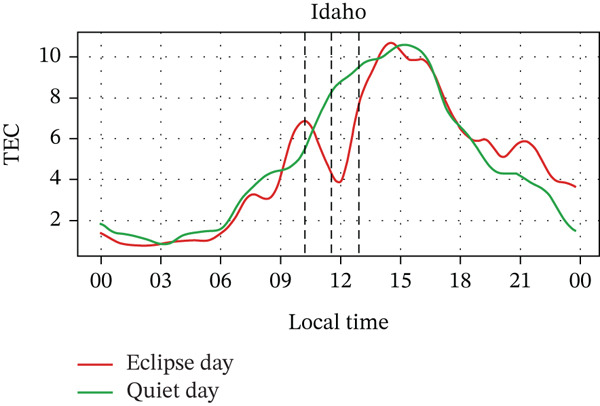
(e)

Figure 6 shows a comparison of the changes in the maximum ionization height (hmF2) during quiet days and the eclipse day. In every station, hmF2 is higher during the eclipse compared to the quiet day. The Idaho and Boulder stations (which are situated near the path of totality) show larger changes compared with the other stations away from the totality. However, unlike foF2 and TEC, there is no sharp variation in hmF2 during the eclipse. The difference in hmF2 between the eclipse day and the quiet day is clearly noticeable, but there is no peculiar variation during the eclipse duration.

Figure 6Changes in maximum ionization height (hmF2) in kilometers recorded at different stations, (a) Alpena, (b) Austin, (c) Boulder, (d) Eglin, and (e)Idaho, during total solar eclipse of 21 August 2017. The three vertical black lines indicate the beginning (left line) and the end (right line) of partial eclipse and maximum (middle line) of the solar eclipse.

**Figure:**
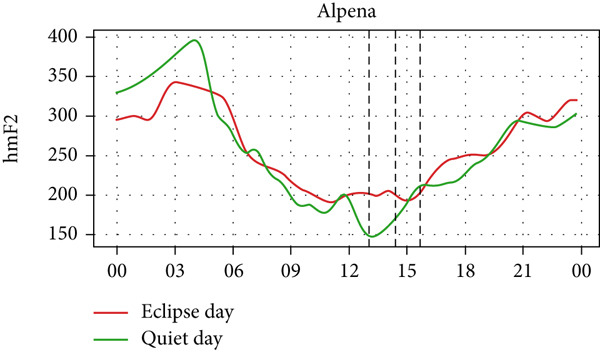
(a)

**Figure:**
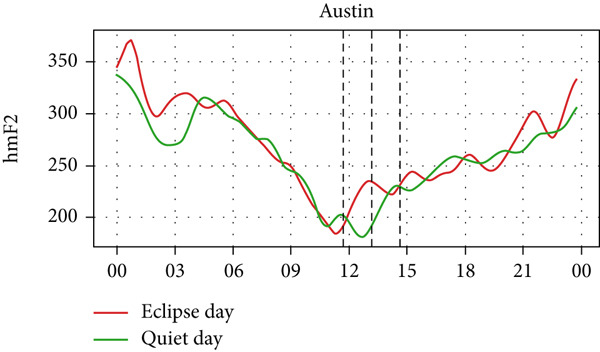
(b)

**Figure:**
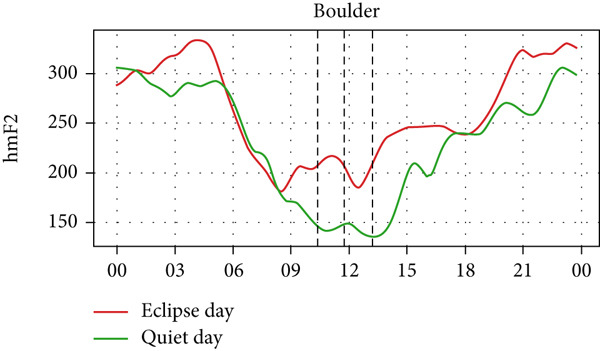
(c)

**Figure:**
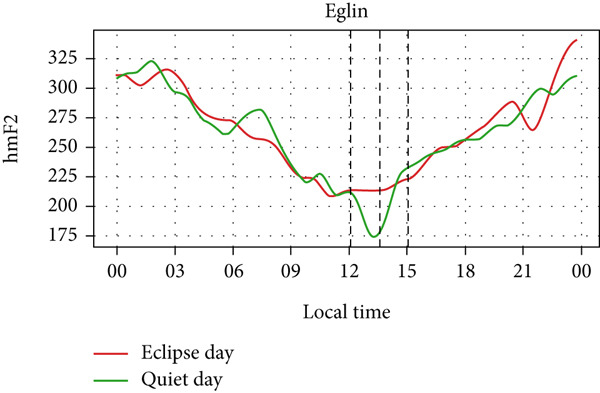
(d)

**Figure:**
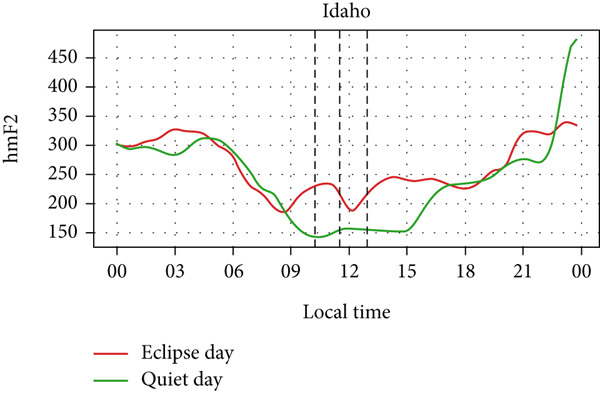
(e)

### 3.2. Event 2: 20–21 May 2012

**Figure 7:**
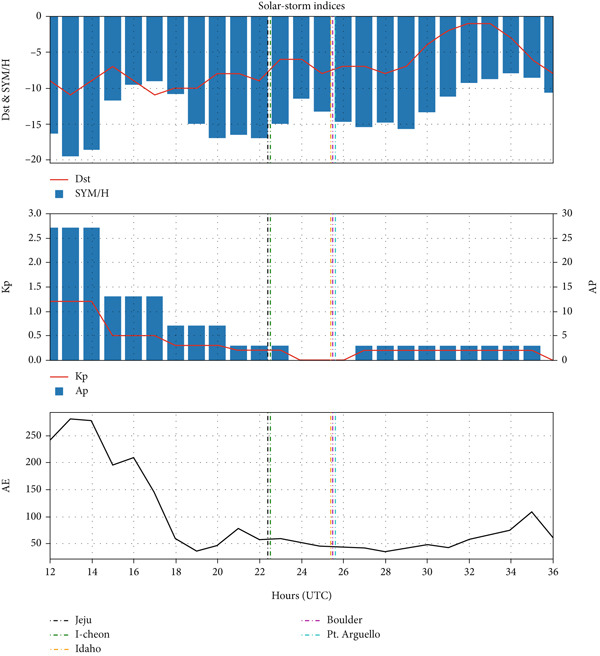
Variation in Dst (nT) and SYM/H (nT) indices in the top panel, Kp and Ap (nT) indices in the middle panel and AE index (nT) in the bottom panel during annular solar eclipse of 20–21 May 2012 (latter half of 20 May and earlier half of 21 May). The vertical lines denote the maximum time of the solar eclipse at each station.

Figure 7 shows the geomagnetic indices during the annular solar eclipse of 20–21 May 2012. The plots have clearly shown that Dst and SYM/H indices were at their minimum during the main phase of the eclipse rather than at the fore or later hours of the eclipse. Considering the very low magnitudes of the AE index and Kp index, the day was a geomagnetically quiet day, which provides an opportunity to study the ionospheric responses to the eclipse.

Figure 8 depicts the variation of foF2 at four different stations: Boulder, I‐cheon, Idaho, and Jeju. Two of the stations lie in Asia, whereas two of them lie in North America. So, the eclipse occurred on two different days: the evening of May 20 (LT) in Idaho and Boulder and the morning of May 21 (LT) in I‐Cheon and Jeju. In the second and fourth panel, which shows the temporal evolution of rising foF2 in I‐Cheon and Jeju, a dip can be noticed in Jeju as the partial eclipse begins, whereas there is rise in I‐Cheon. In Jeju, foF2 drops from ∼8 MHz to ∼6.2 MHz compared with a quiet day. In I‐Cheon it rises to ∼7.9 MHz compared with ∼6.9 MHz on a quiet day. In the first, and third panels, the lowering of foF2 is significant even though the sun had already reached near the horizon. The dip is noticeable, with foF2 dropping from ∼7 MHz to ∼6.2 MHz in Boulder and ∼6.7 MHz to ∼5.6 MHz in Idaho both compared with quiet days. However, the enhancement before the eclipse is not noticeable in Boulder, Idaho and Jeju stations. Even though there are similar magnitudes of fluctuations throughout the eclipse day as well as on a quiet day, the sudden dip during the eclipse is consistent with only three stations except for I‐Cheon. Hence, this sudden fall in foF2 is attributed to the solar eclipse.

Figure 8Changes in F2‐region critical frequency (foF2) in MHz during the annular solar eclipse of 20 May 2012 (LT) at (a) Boulder and (c) Idaho and 21 May 2012 (LT) at (b) I‐Cheon and (d) Jeju. The three vertical black lines indicate the beginning (left line) and the end (right line) of the partial eclipse and maximum (middle line) of the solar eclipse. In Boulder and Idaho, the right vertical line indicates sunset.

**Figure:**
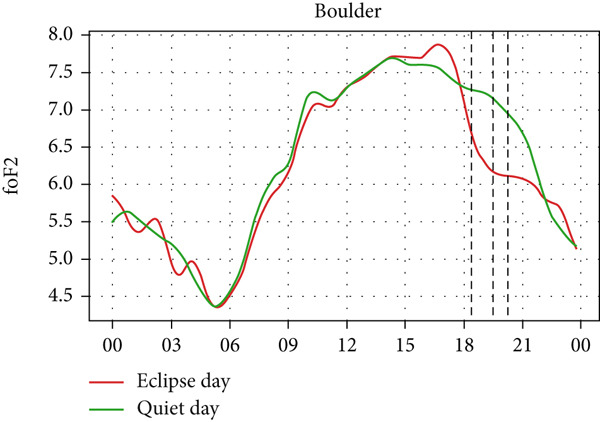
(a)

**Figure:**
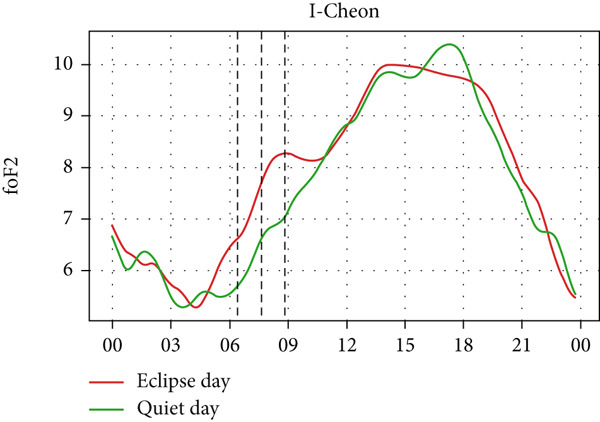
(b)

**Figure:**
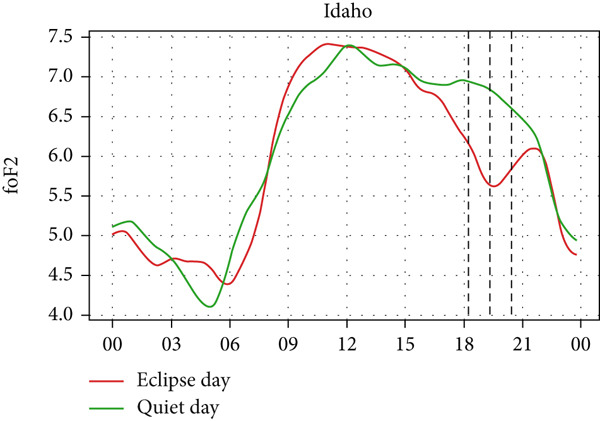
(c)

**Figure:**
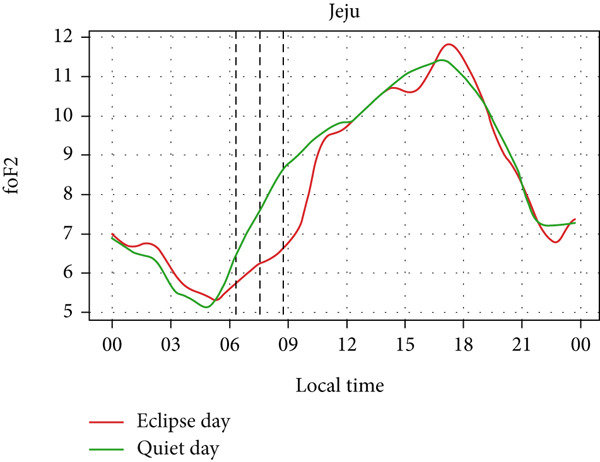
(d)

A similar effect can be seen in Figure 9 where a sudden dip in TEC is observed during the eclipse event as also shown by Silwal et al. [41], which showed 1–4 units of decrease in TEC. The lowering of the TEC curve due to the eclipse blends in with the decreasing phase of the daily cycle of TEC variation in Boulder and Idaho as the sunset occurs during the partial eclipse in Boulder and just after the eclipse in Idaho. During the evening, the low intensity of the Sun as its rays make low angles with the Earth′s surface results in a lowering of foF2 and TEC. This phenomenon, along with solar eclipse, decreases the level of foF2 and TEC, even though we see a decrease in Boulder, whereas an increase in Idaho. Whereas in I‐Cheon, TEC drops from ∼17.5 × 10^16^ m^−2^ to ∼12.5 × 10^16^ m^−2^, and in Jeju, it drops from 20 × 10^16^ m^−2^ to 15 × 10^16^ m^−2^ compared with quiet day. This drop in TEC is similarly attributed to the solar eclipse.

Figure 9Changes in TEC in (×10^16^ m^−2^) during the annular solar eclipse of 20 May 2012 (LT) at (a) Boulder, and (c) Idaho and 21 May 2012 (LT) at (b) I‐Cheon and (d) Jeju. The three vertical black lines indicate the beginning (left line) and the end (right line) of the partial eclipse and the maximum (middle line) of the solar eclipse. In Boulder and Idaho, the right vertical line indicates sunset.

**Figure:**
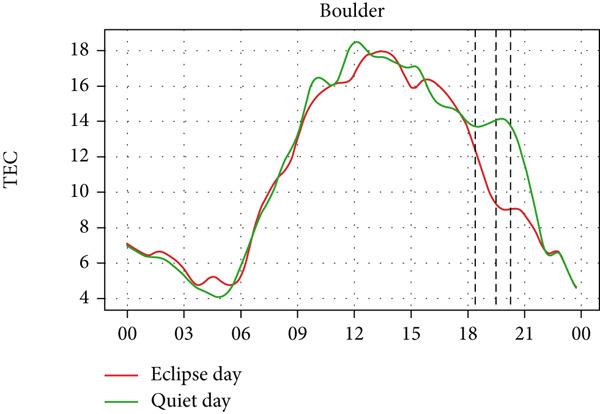
(a)

**Figure:**
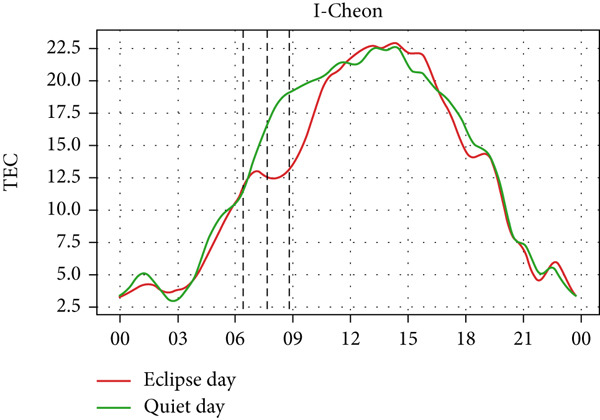
(b)

**Figure:**
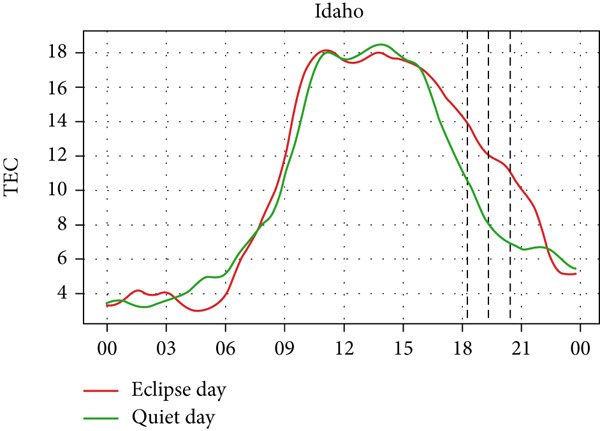
(c)

**Figure:**
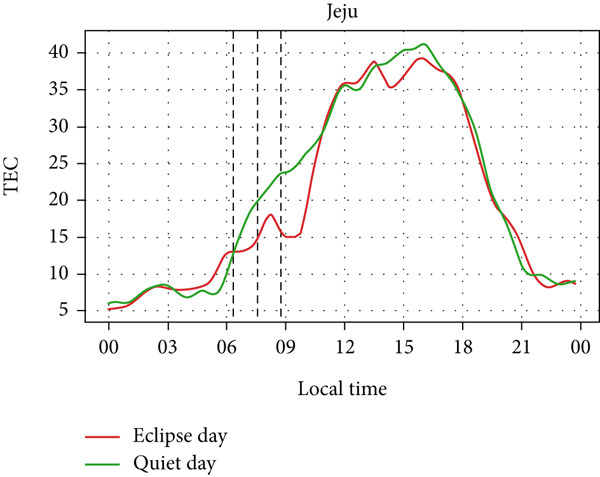
(d)

However, hmF2 in Figure 10 did not show any significant fluctuations as shown in the previous event, as no significant number of fluctuations can be seen in the red curve during the eclipse. This result corresponds with that of Chuo [14], who also found no substantial variation in hmF2. These observations show that the annular solar eclipse has its effects on the ionosphere. However, its effects are not as significant as that of the total solar eclipse. A reasonable interpretation is that although foF2 and TEC respond rapidly to reduced ionization, hmF2 remains relatively stable due to its dependence on slower, altitude‐regulating dynamics that are less affected by the transient shadow of the eclipse. This highlights the ionosphere’s layered behavior, where density and height respond on different timescales and to distinct physical drivers. It is also likely that under conditions of greater eclipse magnitude and totality, such as during the 2017 total solar eclipse, variations in hmF2 may become more evident than other partial eclipses. Finally, solar eclipses, occurring for just a few hours of time, have a noticeable effect on ionospheric parameters, especially on foF2 and TEC.

Figure 10Changes in maximum ionization height (hmF2) in kilometers during the annular solar eclipse of 20 May 2012 (LT) at (a) Boulder, and (c) Idaho and 21 May 2012 (LT) at (b) I‐Cheon and (d) Jeju. The three vertical black lines indicate the beginning (left line) and the end (right line) of the partial eclipse and the maximum (middle line) of the solar eclipse. In Boulder, the right vertical line indicates sunset.

**Figure:**
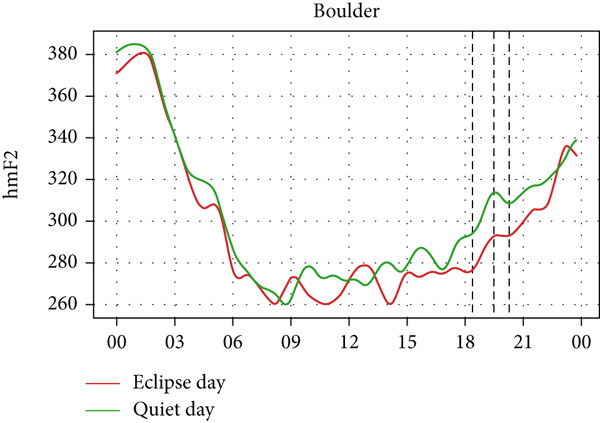
(a)

**Figure:**
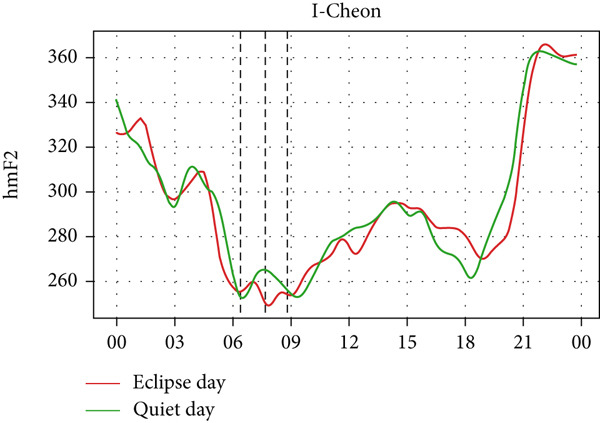
(b)

**Figure:**
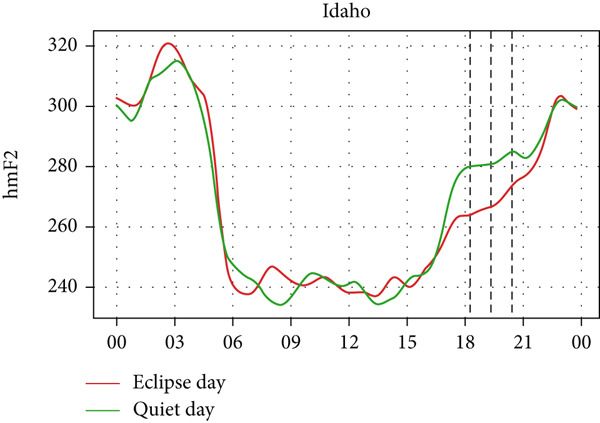
(c)

**Figure:**
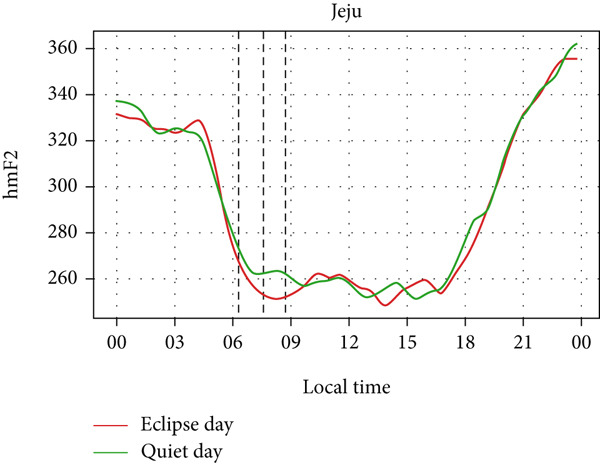
(d)

## 4. Conclusions

In this work, we studied the ionospheric responses to the 21 August 2017 total and 20–21 May 2012 annular solar eclipses, focusing on foF2, TEC, and hmF2 across midlatitude stations. Both eclipses induced significant perturbations in ionospheric parameters, driven by the abrupt reduction of solar EUV radiation, which is critical for ionospheric plasma production. Key findings of our study are summarized below:

• The comparative analysis of both annular and total solar eclipses at midlatitude stations shows the pronounced reductions in foF2 and TEC (30%–40%), exceeding the annular eclipse′s effects (15%–25% declines), consistent with previous findings [4, 25]. Stations under totality (e.g., Idaho, 100% obscuration) exhibited the most severe depletions, indicating the positive relationship between obscure magnitude and ionospheric response. The total eclipses induce significantly stronger hmF2 uplift (~20 km) due to pronounced thermospheric cooling, whereas annular eclipses show negligible hmF2 changes. The recovery times for foF2 (1–3 h) consistently exceed TEC restoration, highlighting the F2‐layer′s dependence on slow transport processes (ambipolar diffusion and neutral winds) rather than photochemical equilibrium alone [38].
• Although transient pre‐eclipse enhancements in foF2 and TEC were observed at some stations, this study did not perform dedicated spectral or wave analysis techniques (e.g., spectral decomposition and Hovmöller diagrams) to confirm the presence of eclipse‐induced TIDs or AGWs. Recent studies (e.g., [42]) have highlighted that filtering techniques can introduce artificial wave‐like features, potentially leading to misinterpretation. Therefore, we acknowledge this limitation and refrain from drawing firm conclusions regarding the presence of eclipse‐induced TIDs or AGWs based on our data. Additionally, recent research [43, 44] has emphasized that occultation geometry, particularly the moments when the umbra and penumbra cones first contact the Earth′s atmosphere, plays a significant role in modulating ionospheric responses, even before visible obscuration begins. These geometric effects may help explain some of the pre‐eclipse variations observed in our study, offering a plausible alternative to wave‐based interpretations. Future work could apply dedicated analyses to further clarify the physical drivers behind such observations.
• The altitude of peak electron density (hmF2) responded distinctly to each eclipse type. During totality, hmF2 rose by ~20 km, which might be due to thermospheric cooling (> 100 K) and subsequent wind‐driven plasma uplift [25]. Conversely, the annular eclipse, with partial EUV reduction, produced negligible hmF2 changes, as weaker cooling insufficiently perturbed transport dynamics [14]. This discrepancy suggests the sensitivity of vertical plasma motion to eclipse‐driven thermospheric forcing.
• Midlatitude stations (Idaho and Boulder) experienced stronger depletions than lower latitudes (Eglin and Austin), reflecting the latitudinal gradient in solar zenith angle effects [31]. However, we note that these midlatitude stations also lie in the high obscuration region. Equatorial regions, though not studied here, likely exhibit amplified responses due to the Appleton anomaly [37]. Eclipses occurring near sunset compound depletions with diurnal plasma decay, masking eclipse‐specific effects. Morning/evening eclipses showed minimal deviations from daily variations, as low solar elevation angles inherently limit ionization, which is consistent with Le et al. [12, 13].

The observed asymmetry between rapid plasma loss and prolonged recovery challenges assumptions of photochemical equilibrium in ionosphere‐thermosphere models (ITMs). Incorporating eclipse‐specific forcing, particularly wind‐driven transport and AGW modulation, is critical for improving predictive accuracy during transient solar events [30]. These findings also bear practical relevance for satellite communication and GNSS systems, where eclipse‐induced TEC fluctuations can degrade signal integrity [20]. Future work could expand geographic coverage to equatorial and high‐latitude regions to resolve latitudinal disparities in eclipse responses. Multi‐instrument campaigns, combining ionosondes, GNSS, and incoherent scatter radars, could disentangle photochemical and transport processes. Additionally, the use of microelectronics and semiconductor technology [45–47] in ionospheric research could be utilized to enhance data collection, signal processing, and real‐time analysis. By integrating advanced semiconductor sensors, AI‐driven processing, and adaptive communication systems, we can achieve higher accuracy in ionospheric parameter measurements and better isolate eclipse‐induced changes from other geomagnetic phenomena.

## Conflicts of Interest

The authors declare no conflicts of interest.

## Author Contributions

Manghang Limbu, Samyam Pudasaini, Sujan Prasad Gautam, Binod Adhikari, Ashok Silwal, Iva Kumari Lamichhane, Rohit Bhattarai, and Pitri Bhakta Adhikari contributed equally to this work.

## Funding

No funding was received for this manuscript.

## Data Availability

The data that support the findings of this study are available on request from the corresponding author. The data are not publicly available due to privacy or ethical restrictions.
